# Clinical and epidemiological characteristics of cutaneous leishmaniasis in Sri Lanka

**DOI:** 10.1186/s12879-018-2999-7

**Published:** 2018-03-06

**Authors:** Devika Iddawela, Sanura Malinda Pallegoda Vithana, Dhilma Atapattu, Lanka Wijekoon

**Affiliations:** Department of Parasitology, Faculty of Medicine, University of Peradeniya, Peradeniya, Sri Lanka

**Keywords:** Cutaneous Leishmaniasis, Sri Lanka, *Leishmania donovani*

## Abstract

**Background:**

Leishmaniasis, a vector borne tropical/subtropical disease caused by the protozoan *Leishmania* is transmitted to humans by sandfly vectors *Phlebotomus* and *Lutzomyia*. The principal form found in Sri Lanka is cutaneous leishmaniasis (CL) and is caused by *Leishmania donovani*. A rising trend in disease prevalence has been observed recently in Sri Lanka and the island is in fact the newest endemic focus in South Asia. Determining the prevalence of smear positivity among clinically suspected CL patients, identifying risk factors and specific clinical presentations of CL in order to implement preventive and early treatment strategies were the objectives of this study.

**Methods:**

A sample of 509 clinically suspected cases of CL referred to the Department of Parasitology from all across Sri Lanka between 2005 and 2015 was selected consecutively. Diagnosis was confirmed by microscopic visualization of the *Leishmania* amastigote from the slit skin smear. A structured questionnaire was used to identify exposure related risk factors and a clinical examination was performed to identify lesion characteristics.

**Results:**

Out of 509 clinical cases, 41.5% (*n* = 211) were smear positive. The study population ranged from ages 1 to 80 years (mean age = 34.76) and the most affected age group was 40–49. Of the smear positives, 58.85% were males. Majority (47.86%) were from the North Western region (Kurunegala) of the country and were exposed to scrub jungles. Sand fly exposure (*p* = 0.04) and positive contact history (*p* = 0.005) were significant risk factors for smear positivity.

Erythema (*p* = 0.02), lack of pruritus (*p* = 0.02) and scaly appearance (*p* = 0.003) were significant lesion characteristics in smear positivity. Lesions were commonly found in the exposed areas and the commonest morphological type was papulo-nodular.

**Conclusions:**

An increasing trend in the spread of cutaneous leishmaniasis from endemic to non-endemic areas has become evident. Positive contact history and sandfly exposure were significant risk factors for smear positivity which may indicate the possibility of human reservoir hosts in infection transmission. Lack of pruritus, scaly appearance and erythema were highly significant lesion characteristics associated with *Leishmania* positive smears which can be used for the clinical diagnosis of CL.

## Background

Leishmaniasis, a vector borne tropical/subtropical disease caused by the protozoan belonging to the genus *Leishmania*is transmitted to humans by sandflyvectors*Phlebotomus* and *Lutzomyia*. The clinical manifestations of the disease vary from cutaneous, muco-cutaneous to visceral leishmaniasis (VL) and the severity depends on the *Leishmania* species involved and the type of immune response to infection [1]. Leishmaniasis is a disease affecting about 89 countries and is endemic to Asia, Africa, parts of North and South America and the Mediterranean. Globally it is thought that 12 to 15 million are infected; with 1 to 2 million new cases and about 70,000 deaths reported yearly [1]. About 350 million worldwide are at risk of acquiring the diseasewhich has made it one of the seven most important tropical diseases according to the World Health Organization (WHO) [1].

In the Old World (Asia, Africa and Mediterranean littorals), CL is caused by *Leishmania major, L. tropica* and rarely*, by L. infantum* and *L. donovani* [2] and in the New World CL is caused by either in the *L. Mexicana* species complex (*L. mexicana*, *L. amazonensis*, and *L. venezuelensis*) or the subgenus *Viannia* (*L. [V.] braziliensis*, *L. [V.] guyanensis*, *L. [V.] panamensis,* and *L. [V.] peruviana*) [3].

In Sri Lanka the predominant form is cutaneous leishmaniasis (CL) and is suprisingly caused by *L. donovani* zymodeme MON – 37 which is also responsible for VL in East Africa and India [4].

Cutaneous lesions of CL are frequently found in exposed areas of the body: forearms, legs and face where sandfly bites occur most often. Initially a small erythematous papule maybe visible at the bite site and over 4 to 12 weeks the lesion assumes a nodular-plaque like appearance and gives off a seropurulent discharge. This later dries up and then ulcerates giving a “volcanic” nodulo-ulcerative appearance which is said to be classical for CL [5]. Some lesions can develop into other morphological forms such as eczematoid, warty, plaque, zosteriform, hyperkeratotic and erysipeloid. These lesions are usually painless unless secondarily infected and regress over a period of 2 to 12 months [5].

Visualization of parasite by direct microscopy of lesion smears is considered a gold standard diagnostic method. Direct microscopy together with the clinical and epidemiological characteristics, provide the basis for diagnosing CL in Sri Lanka. Apart from this, PCR is emerging as the most accurate method of parasitological diagnosis [6, 7].

Leishmaniasis is a disease affecting about 89 countries and is endemic to Asia, Africa, parts of North and South America and the Mediterranean. Globally it is thought that 12 to 15 million are infected; with 1 to 2 million new cases and about 70,000 deaths reported yearly [1]. About 350 million worldwide are at risk of acquiring the diseasewhich has made it one of the seven most important tropical diseases according to the World Health Organization (WHO) [1].

The first reported case of leishmaniasis in Sri Lanka was from 1990 however this was speculated to be due to overseas employees returning to Sri Lanka [8]. The first case due to local transmission was reported in 1992and since then, leishmaniasis has become well established in Sri Lanka with an increasing number of cases and distribution across the country [3, 8, 9]. All reported cases were CL until 2005 when the 1st case of muco-cutaeous leishmaniasis (MCL) was reported. Only 2 years later in 2007, the first VL case was reported [10, 11]. In 2008, the Epidemiology Unit of Sri Lanka named leishmaniasis as one of the notifiable diseases in the country. Sri Lanka is now considered endemic and is the newest focus of leishmaniasis in South Asia [8]. The sandfly, *Phlebotomus argentipes*, is the natural vector of *L. donovani* and vector studies have shown that all three members of the Phlebotomus complex, *Phlebotomus glaucus, Ph. Argentipes* sensu stricto *and Ph. Annandalei* are prevalent in Northern Sri Lanka [12]. In 2011, Senanayake et al.*,* [13], were able to prove Phlebotomus as the vector of leishmaniasis in Sri Lanka.

With deforestation, changing lifestyles and environmental conditions in the island, the sandfly population and its contact with humans is expected to increase which may result in outbreaks of the disease [8]. Most of the studies carried out in Sri Lanka were from North Central and Southern provinces [3, 14]. There was pausity of data regarding CL in North Western and Central provinces. Therefore this study was conducted to fill the gap in knowledge related to this important infection in other areas of Sri Lanka. The objectives of this study were to determine the prevalence of smear positivity among clinically suspected CL patients, find out factors associated with smear positivity and to identify common clinical manifestations of the disease.

## Methods

### Study design and data collection

A total of 509 clinically suspected cases of CL referred to the Department of Parasitology, Faculty of Medicine, Peradeniya from all over Sri Lanka between 2005 and 2015 were consecutively selected for the study. Ethical clearance was obtained from the Ethics Review Committee of the Faculty of Medicine, University of Peradeniya. Study objectives, goals and procedure of sample collection were explained both verbally and in writing in their native languages (Sinhala and Tamil) and informed written consent was obtained. Patient information and samples were kept confidential at all times. All tested samples were disposed appropriately according to local policy.

Sociodemographic information such as age, sex, district of residence, occupation and exposure related risk factors such as exposure to scrub jungles, presence of sandflies in the environment, contact history, comorbidities (Diabetes Melitus, Cardiovascular disease, Hypertension, COPD/Asthma or any other chronic illness) and overseas travel within 1 year were gathered from patients. A thorough clinical examination along with a history was obtained to identify lesion characteristics: site, size, duration of lesion, number of lesions (including satellite lesions), presence of inflammatory signs, type and appearance of lesion. The lesions were categorized into papulo-nodular, nodulo-ulcerative, ulcerative and plaque depending on their appearance. A solid, elevated lesion with no visible fluid and a diameter of > 0.5cmwas categorized as a papulo-nodular lesion. Small, glistening, translucent skin over a colored papule with a central ulcer and raised pearly edges was considered as nodulo-ulcerative. Discontinuation of the epithelial lining extending into the dermis was categorized under ulcerative lesions. A plaque was defined as a raised, flat topped lesion > 1 cm.Lesions were further categorized into wet and dry lesions. Those with sero-purulent exudates and inflamed open margins were considered wet lesions and those covered by crust and scab were categorized as dry lesions.

### Laboratory diagnosis

A slit skin smear was performed in each participant under sterile conditions. The skin was cleaned with 70% alcohol. A sterile lancet was then used to obtain tissue fluid from 3 sites at the edges of the skin lesion.Smears were made on sterile slides, air dried, fixed with methyl alcohol for 30 s, stained with 10% Giemsa and observed for *Leishmania* amastigotes under the light microscope. The smear was considered negative if no *Leishmania* amastigotes were visible at × 100 magnification under oil immersion field. Each slide was examined by two trained laboratory technologists to improve the accuracy of the test.

### Data analysis

Data obtained was entered on to an excel spreadsheet and transferred to SPSS version 21 statistical program. *Leishmania* smear positivity was calculated as the number of smear positive subjects among the study group. Bivariate analysis using the Chi-square test was used to calculate the associations between the documented risk factors and smear positivity. Those with a *p* value < 0.05 were considered significant.

## Results

### Socio-demographic characteristics of study population

A total of 509 subjects participated in this study. The age distribution ranged from 1 year to 80 years with a mean of 34.76 and a standard deviation of 18.563. Males (55%) outnumbered females (45%) in the study population.Cases were from 12out of the 25 districts in Sri Lanka with most being referredfrom Kurunegala (*n* = 245 or 48.5%) followed by Matale (*n* = 99 or 19.6%) and Kandy (*n* = 87 or 17.2%). Of the participants, 30.7% were unoccupied, 21% were school going children, 10% belonged to armed forces and 8.2% were farmers.Of the study population, 75.9% and 75.7% were exposed to scrub jungles and sandfly bites respectively. Insect repellents were used on a regular basis by 12.4%. Past history of similar clinical features were reported by 6.5%.

### Demographic and other factors associated with smear positivity

**Fig. 1:**
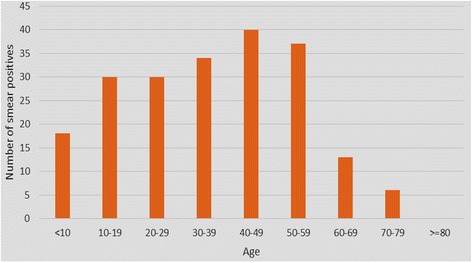
Smear positivity among age groups

**Fig. 2:**
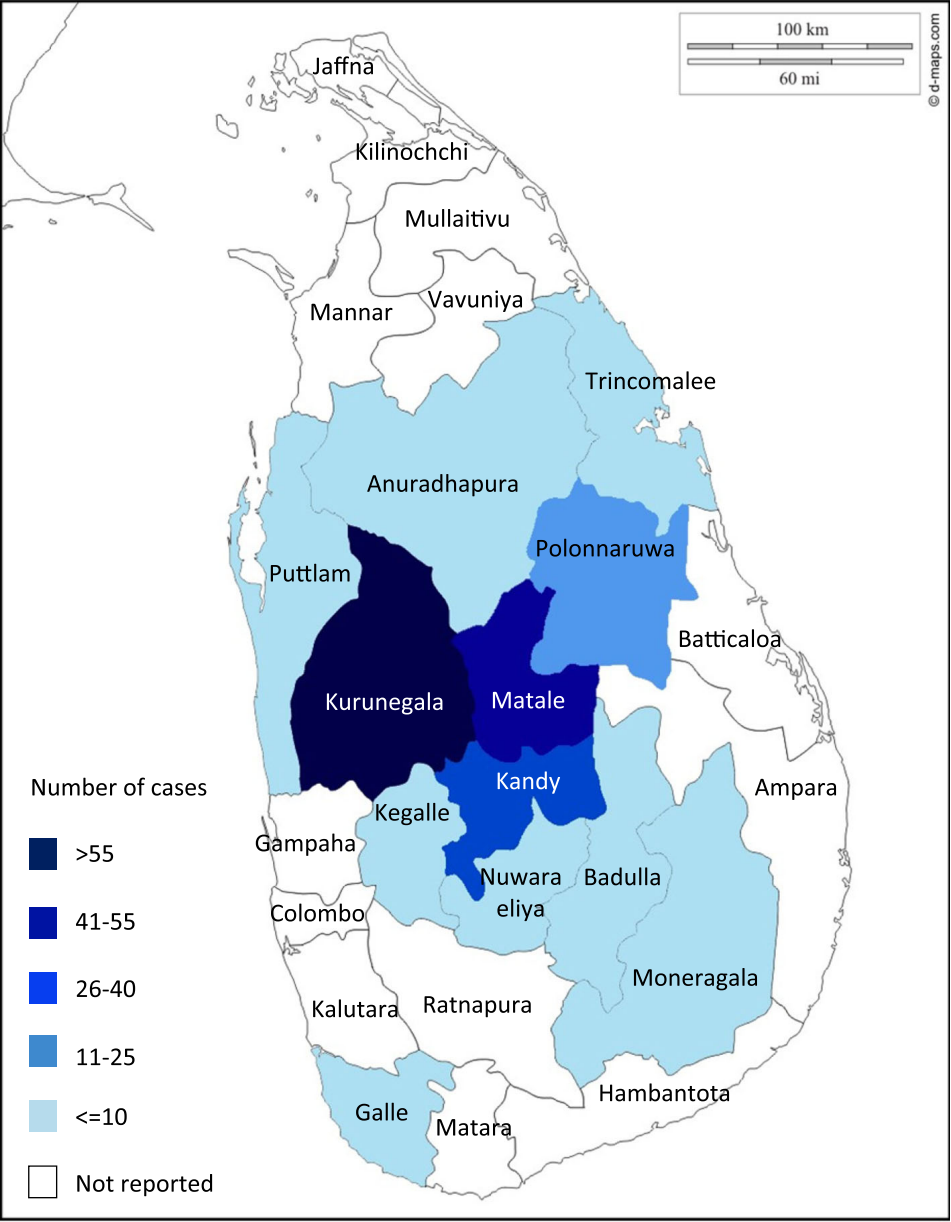
Geographical representation of smear positive cases

**Fig. 3:**
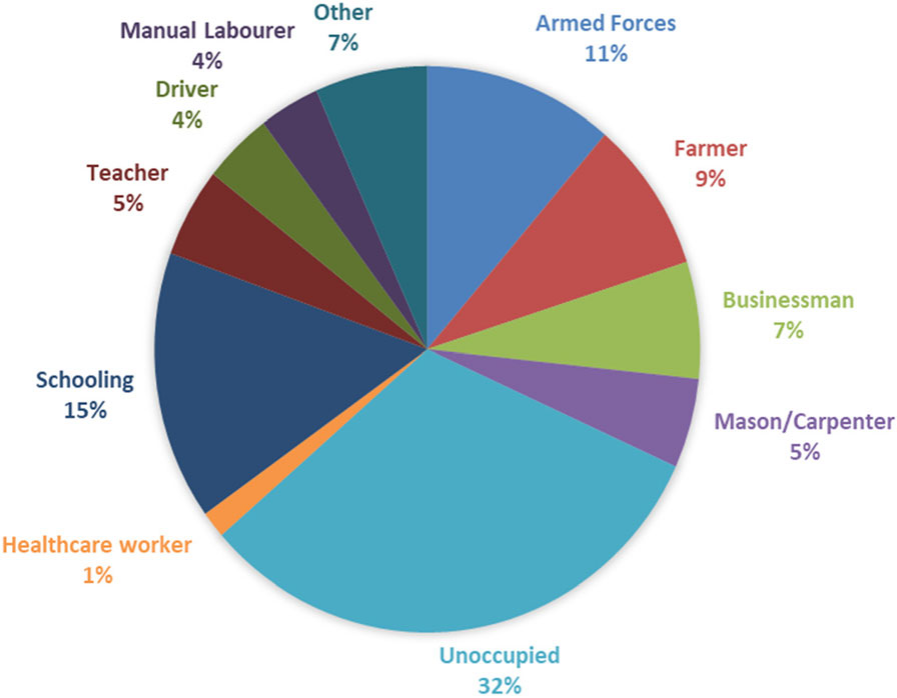
Smear positivity according to occupation

Of the total study population, 41.5%(*n* = 211) were smear positive.Males had a higher (44.4%) smear positivity for *Leishmania* amastigotes compared to females (37.9%). The highest age specific smear positivity (50.7%) was observed in those between 40 and 49 years of age (Fig. 1). However smear positivity was not significantly different by age or sex of the study participant. Most smear positive cases were from Kurunegala (*n* = 101) followed by Matale (*n* = 44) and Kandy (*n* = 31) districtsand interestingly one positive case was reported from Nuwaraeliya district (Fig. 2). Majority of those with positive smears were unoccupied (Fig. 3). Out of the exposure related risk factors analysed, the presence of sandflies in the environment (*p* = 0.04) and contact history of disease (*p* = 0.005) were highly significant risk factors whereas exposure to scrub jungles was not, even though a large number of smear positives were exposed to these. Past history of similar cutaneous manifestations, presence of comorbidities such as diabetes, hypertension, COPD/asthma, ischaemic heart disease and recent travel to other *Leishmania* endemic regions were not significant risk factors for smear positivity (Table 1).

**Table 1:** Smear positivity with demographic and exposure related risk factors

Demograhic and other Risk Factors	Smear positive	Smear negative	Total	Chi^2^	*p* Value
	Number / (%)	Number / (%)			
Sex (*n* = 504)				2.184	0.139
Male	123 (44.4)	154 (55.6)	277		
Female	86 (37.9)	141 (62.1)	227		
Presence of scrub jungle near residence (*n* = 460)				0.708	0.4
Yes	151 (43.3)	198 (56.7)	349		
No	43 (38.7)	68 (61.3)	111		
Presence of sandflies in environment (*n* = 473)				4.412	0.04
Yes	158 (44.1)	200 (55.9)	358		
No	38 (33)	77 (67)	115		
Contact history (*n* = 508)				7.86	0.005
Yes	25 (62.5)	15 (37.5)	40		
No	186 (39.7)	282 (60.3)	468		
Past history of similar cutaneous manifestations (*n* = 367)				2.262	0.133
Yes	6 (25)	18 (75)	24		
No	139 (40.5)	204 (59.5)	343		
Comorbidities (*n* = 176)				0.038	0.845
Yes	19 (35.8)	34 (64.2)	53		
No	46 (37.4)	77 (62.6)	123		
Travel abroad within 1 year (*n* = 448)				0.338	0.561
Yes	16 (45.7)	19 (54.3)	35		
No	168 (40.7)	245 (59.3)	413		

### Clinical features and lesion characteristics in relation to smear positivity

Most of the lesions were observed in exposed parts of the body of which the head and neck region was the most affected in terms of the number of lesions. Site specific smear positivity was highest in the trunk with 53.1% returning positive smears. The site of lesion however was not found to be statistically significant (*p* = 0.306). Lack of pruritus (*p* = 0.017), presence of erythema (*p* = 0.023) and scaly appearance (*p* = 0.003) were lesion characteristics which had statistically significant associations with smear positivity.

Out of the lesions that were smear positive, most lasted less than 6 months (54.5%), were papulo-nodular (43.9%) in type and were painless (89.8%). Satellite lesions were absent in the majority (93.6%). The highest smear positivity was seen in lesions measuring 15-29 mm (45.3%). These as well as clinical signs such as fever, local lymphadenopathy and hypo/hyperpigmentation were not associated significantly with positive smears for *Leishmania* amastigotes (Table 2).

**Table 2:** Lesion characteristics and other clinical features

Lesion characteristics/ Clinical features	Smear positive	Smear negative	Total	Chi^2^	*p* Value
	Number / (%)	Number / (%)			
Duration (*n* = 491)				0.626	0.731
< 6 months	110 (42.5)	149 (57.5)	259		
6–12 months	47 (38.2)	76 (61.8)	123		
> 12 months	45 (41.3)	64 (58.7)	109		
Site of Lesion (*n* = 497)				3.612	0.306
Head and Neck	114 (43.5)	148 (56.5)	262		
Trunk	17 (53.1)	15 (46.9)	32		
Upper Limbs	57 (37.7)	94 (62.3)	151		
Lower Limbs	19 (36.5)	33 (63.5)	52		
Size of Lesion (*n* = 436)				3.139	0.371
< 15 mm	82 (39.4)	126 (60.6)	208		
15-30 mm	68 (45.3)	82 (54.7)	150		
30-45 mm	20 (44.4)	25 (55.6)	45		
> 45 mm	10 (30.3)	23 (69.7)	33		
Multiple Lesions (*n* = 506)				3.272	0.07
Yes	47 (50)	47 (50)	94		
No	164 (39.8)	248 (60.2)	412		
Satellite lesions (*n* = 491)				0.146	0.703
Present	13 (38.2)	21 (61.8)	34		
Absent	190 (41.6)	267 (58.4)	457		
Type of lesion (*n* = 468)				1.814	0.612
Papulo-nodular	87 (46)	102 (54)	189		
Nodulo-ulcerative	45 (40.2)	67 (59.8)	112		
Ulcerative	41 (39.4)	63 (60.6)	104		
Plaque	25 (39.7)	38 (60.3)	63		
Pruritus (*n* = 493)				5.696	0.017
Present	37 (32.2)	78 (67.8)	115		
Absent	169 (44.7)	209 (55.3)	378		
Hypopigmentation (*n* = 324)				1.992	0.158
Present	53 (44.5)	66 (55.5)	119		
Absent	75 (36.6)	130 (63.4)	205		
Hyperpigmentation (*n* = 231)				0.823	0.364
Present	32 (38.1)	52 (61.9)	84		
Absent	65 (44.2)	82 (55.8)	147		
Erythema (*n* = 389)				5.148	0.023
Present	101 (46.1)	118 (53.9)	219		
Absent	59 (34.7)	111 (65.3)	170		
Dry Lesion (*n* = 276)				2.870	0.09
Yes	98 (42.4)	133 (57.6)	231		
No	13 (28.9)	32 (71.1)	45		
Scaly lesion (*n* = 276)				8.584	0.003
Yes	90 (45.2)	109 (54.8)	199		
No	20 (26)	57 (74)	77		
Pain (*n* = 493)				2.023	0.155
Present	21 (33.3)	42 (66.7)	63		
Absent	184 (42.8)	246 (57.2)	430		
Fever (*n* = 468)				0.059	0.807
Present	8 (44.4)	10 (55.6)	18		
Absent	187 (41.6)	263 (58.4)	450		
Local Lypmphadenopathy (*n* = 376)				0.154	0.695
Present	20 (40)	30 (60)	50		
Absent	140 (42.9)	186 (57.1)	326		

## Discussion

Sri Lanka is considered an endemic country despite the fact that documentation of leishmaniasis started merely two decades ago [8]. An overall smear positivity of 41.5% was found among the study population. Similar studies conducted in Sri Lanka with clinically suspected CL patients have reported smear positivity of 60.2% and 66.7% [2, 10]. A notable limitation of these studies was the small sample size. Direct microscopy is considered as the gold standard because of its high specificity [15]. However some others have reported PCR diagnosis as approaching gold standard levels [16] Faber et al. have reported low and variable sensitivity in culturing the parasite [17]. Sensitivity for microscopic detection has been shown to vary between 53.98% [18] to 79% [19–21]. Therefore, it can be inferred that the reason for low overall positivity observed in the present study is due to low sensitivity of the diagnostic test, lapses in sample collection and/or laboratory technique and clinical over diagnosis. A limitation of this study was the inability to perform PCR on smear negative samples and therefore early diagnosis of clinically suspected cases with highly sensitive diagnostic tests (PCR) would enable early diagnosis and treatment, thereby preventing spread of infection. Slit skin smear which was repeated in 2 weeks, in selected smear negative patients (with high clinical suspicion) gave positive results.. Therefore, it could be argued that the test would yield higher overall positivity if all negative samples were re-tested. PCR has high sensitivity approaching 100%. Males were affected more than females, which is supported by studies conducted in Sri Lanka and overseas [3, 22, 23]. This may be due to increased exposure to sand fly bites caused by increased time spent outdoors. In Sri Lanka, men tend to be bare-chested while working outdoors whereas those of the opposite sex are well covered, which makes males more prone to insect bites in general. In fact lesions on the trunk were seen almost exclusively in males. A study conducted in Central Amazonia have reported that males were at an increased risk of developing the disease compared to females at similar vector exposure levels. Females were better equipped in containing the CL infection, possibly due to the upregulation of Th1 immune response by oestrogen [24].

Smear positivity was almost uniform from ages 10 through 59, depicting those belonging to the working age group are most affected. Children less than 10 years and elders over 60 years were affected less. This pattern was supported by local studies done in Anuradhapura, Polonnaruwa, Matara in Sri Lanka and in other parts of the world as well [3, 22, 25–28].

Previous Sri Lankan studies have shown CL to be most prevalent in North Western, North Central and Southern regions of the country [3, 14]. This study corroborated this result as it reported the highest prevalence (101/209) from Kurunegala district in the North Western Province. Many of these regions experience extended periods of dry weather and drought on a yearly basis. Scrub jungles are the predominant type of vegetation in these regions and serve as a habitat for sand flies. Human encroachment into areas of scrub jungle increases the exposure to vector bites. In contrast to other local studies, the present study reported considerably high number of CL patients from Matale (44/209) and Kandy (31/209) districts which are in the intermediate and wet zones respectively. Interestingly 1 CL case was reported from the district situated at highest altitude in the island, Nuwara eliya which has an average annual temperature of 15.8 °C [29].A study performed on the effect of temperature on *Leishmania* development in sand flies reported that *L. infantum* and *L. brazilliensis* develop well between 20 °C and 26 °C in majority of sand flies [30]. This may explain why Kandy which has an average annual temperature of 24.5 °C reported a relatively high number of cases. With global warming the spread of vectors into high altitude regions is expected [30]. Thus there is a high possibility of the spread of CL from low country dry zone to wet and intermediate zones in the highlands of Sri Lanka.

Majority of patients who tested positive were unoccupied. This group primarily consisted of housewives who commonly engaged in activities such as gardening and collecting firewood in rural areas. They were followed by school-aged children for highest smear positivity. Children in many of these regions engage in many outdoor activities and are in constant contact with nature. The same observation was made by two studies from Libya and Brazil supporting our results [26, 31]. In contrast to this, a research performed in the North Central Province has reported that military recruits and those engaging in farming had the highest smear positivity [3]. Military personnel are at a greater risk of leishmaniasis because of constant and regular jungle encounters. An incidence of 25% was reported in two studies involving armed soldiers in Belize and the Amazon basin [32, 33]. The number of armed soldiers occupying endemic regions in this country have dramatically reduced since the end of the three decade long civil war and this is reflected by the lower prevalence found among this group in the present study. A significant association between the presence of sand flies in the environment and smear positivity (*p* = 0.04) was evident in the study and is supported by many studies conducted in Sri Lanka and abroad [3, 34].

Most with no contact history of the disease were smear negative. Of those who reported contact, more than half had positive smears. This association was found to be highly significant (*p* = 0.005). In the absence of a known reservoir host in Sri Lanka, CL patients with active lesions can be considered as possible reservoir hosts for the transmission of disease. Previous studies conducted in Sri Lanka have not found a significant association in this aspect [3, 9–15, 35].

The majority of patients in the study had only one lesion and the number of lesions were not significantly associated with smear positivity. However, patients with multiple lesions reported 50% smear positivity whilst those with single lesions reported 38.5% smear positivity. This is in contrast to both local and overseas studies which have reported the highest smear positivity in single lesions [3, 9, 22, 23, 36]. Lack of pruritus (*p* = 0.017), presence of erythema in or around lesion (*p* = 0.023) and scaly appearance (*p* = 0.003) in lesions were highly significant lesion characteristics associated with smear positivity. Further research based on these results can be used to develop a clinical score for the diagnosis of leishmaniasis which would be very useful in initiating treatment in the rural setting.

Many of the lesions were dry and reported greater smear positivity than wet lesions, although the results were not statistically significant. This finding is corroborated by studies done in Sri Lanka and abroad. [3, 9, 37]. Contrary to this, a study conducted in Libya in 2013 reported a significant majority of wet lesions in children and adults with positive smears [26]. A Northern African study has documented that *L. tropica* predominantly causes dry lesions whereas *L. major* causes wet lesions [38, 39]. CL in Sri Lanka is caused by *L. donovani* which gives rise to predominant dry lesions [3]. The similarity in lesions caused by two different species in different geographical regions in the world may indicate similar biological and host immune responses which warrants further research.

The commonest pathological type of lesion associated with positive smears was papulo-nodular, but this was not in agreement with former local and foreign studies. Most of the existing evidence is in favour of nodulo-ulcerative to be the commonest [3, 14, 16, 23, 40]. The type of lesion is thought to depend primarily on the species of *Leishmania* and the immune response mounted by the human body [1, 6, 41].

A study in Yemen has shown that CL has a chronic course and reported high smear positivity between 1 and 6 years [39]. This suggests that *Leishmania* amastigotes are present in the lesion/s until they heal irrespective of the time duration. In contrast to this, a study comparing diagnostic tests for American leishmaniasis has concluded that the sensitivity of smears significantly reduces as lesions become chronic. Sensitivity for acute lesions (< 3 months) being 78.8% and reducing to 44% for chronic lesions (> 12 months). The same study had reported no change in sensitivity when PCR was used and is indeed superior in diagnosis [7]. Even though majority of the cases in the present study were acute (< 6 months), a significant association between smear positivity and the duration of lesion/s was not found.

## Conclusions

There is an increasing trend in the spread of cutaneous leishmaniasis from endemic to non-endemic areas. A significant association between smear positivity and presence of sand flies in the environment and contact history of CL indicate the possibility of human reservoir hosts for transmission of infection. This study emphasises the importance of considering scaly skin lesions with erythema and lack of pruritus along with other risk factors in clinical diagnosis of CL. Active case detection and treatment of CL patients are vital to prevent future outbreaks of the disease.

## Abbreviations

CL: Cutaneous Leishmaniasis
MCL: Muco-cutaneous Leishmaniasis
PCR: Polymerase Chain Reaction
VL: Viesceral Leishmaniasis
WHO: World Health Organization
